# The miseries that remain may yet be alleviated

**DOI:** 10.1073/pnas.2314207121

**Published:** 2024-10-31

**Authors:** Ruben C. Arslan

**Affiliations:** ^a^Wilhelm Wundt Institute for Psychology, Leipzig University, Leipzig 04109, Germany

Killingsworth, Kahneman, and Mellers (1) found a way to reconcile their disagreements about the shape of the relationship between income and happiness: It is log linear on average but flattens among the unhappiest 20% past a threshold of $100,000 (annual gross household income). Kahneman and Deaton (2) did not simply mistake a ceiling effect in their measure for a fundamental truth about money and happiness. Instead, a notoriously tricky phenomenon, heteroskedasticity, confounded both Kahneman and Deaton and Killingsworth (3). Both claims, the linear average effect, and the flattening for the unhappiest 20% can be correct. The (inflation-adjusted) threshold stands.

This new narrative is conciliatory, but only thinly linked to the presented statistical evidence. The existence and location ($100,000) of a threshold was not estimated in Killingsworth’s data but was based on Kahneman and Deaton. Datasets were split at the threshold, then the slopes in the subsets were compared. However, this segmented regression allows a sudden jump in happiness at the threshold, the two slopes do not need to connect at the threshold (Fig. 1). Happiness suddenly increasing at $100,000 among the unhappiest 15% does not fit the narrative and is not mentioned in the paper.

**Fig. 1.**
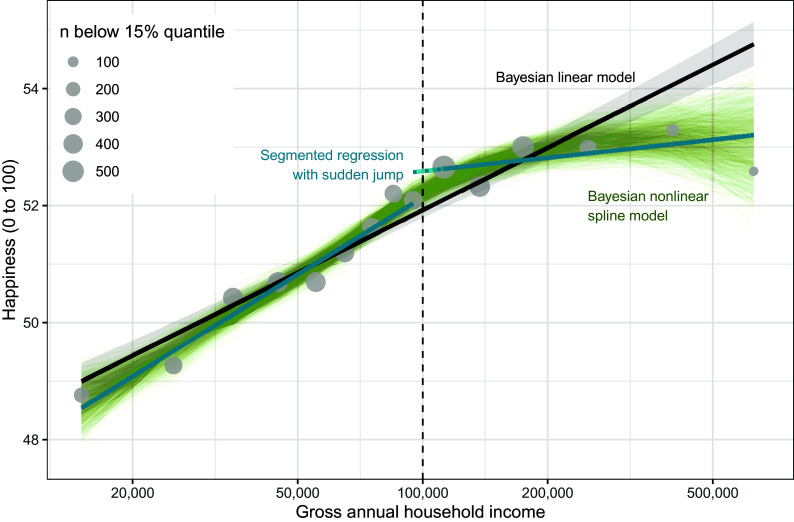
Fitted values for the 15% quantile of happiness according to log income. Three models are shown. In blue, as fit by Killingsworth, Kahneman, and Mellers, a segmented regression allowing for a discontinuity (the gap between the blue line and the dotted blue line). In black, a simple linear quantile regression, which fits well until $200,000 after which data are sparse. In green, draws from a Bayesian nonlinear spline model show a continued linear increase after $100,000 and high uncertainty after $200,000.

I reanalyzed Killingsworth’s aggregated data (the raw data were not released). When I do not build in an assumed threshold and instead freely estimate the shape of the income effect on quantiles, a nonlinear relationship is not clearly superior to a linear relationship in leave-one-out cross-validation (4, 5). Model differences are small relative to inherent uncertainty. So, the evidence for a flattening is not strong. If anything, the spline may flatten only after $200,000, where data are sparse (Fig. 2). Segmented regressions without discontinuities show no significant slope changes at any of the discussed quantiles either. More certainty could come from additional data or from a model of the raw, unaggregated experience sampling data that consider ceiling effects and changes in within-subject variability.

**Fig. 2.**
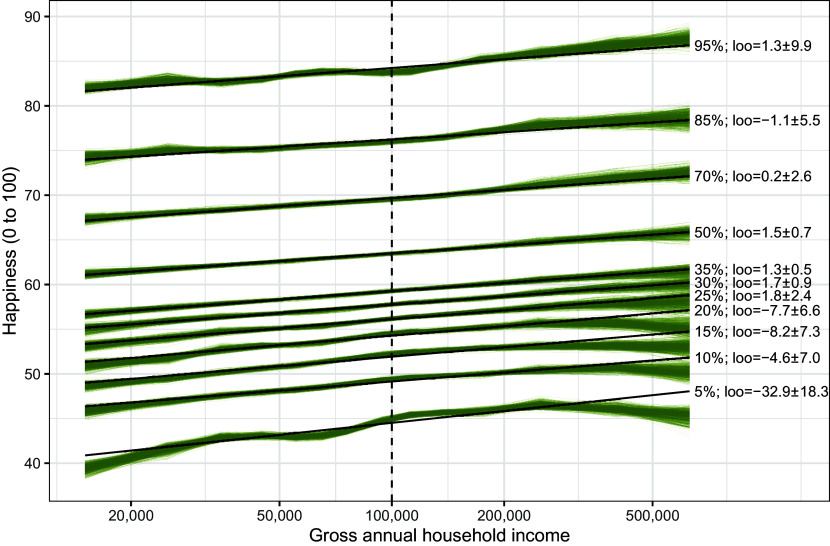
When freely estimated, the spline for the lower quantiles do not flatten before $200,000. Black lines show a linear model’s fit, green lines show a range of possible nonlinear associations using samples drawn from Bayesian quantile spline regressions. Approximative leave-one-out model comparisons show some preference for nonlinearity (negative loo values) at lower quantiles, but all differences are within two SE.

Should the flattening pattern turn out to be robust in further data, would it imply that for a “category of unhappy people” money never improves happiness past a certain threshold? With only a momentary snapshot and little angle to identify a causal effect [unlike (6)], we should consider alternative explanations. Perhaps, at higher incomes, the effect of further income is less *predictable*. Most put the money to good use and improve their happiness; some immiserate themselves by purchasing a social media platform. Still, no one should refuse a raise: the expected value remains positive for everyone, even unhappy people. Or perhaps, more trivially, only disposable, or even discretionary income (“spending money”) increases happiness, not taxes paid. The relationship between gross household income and spending money varies along the income gradient owing to progressive taxation, social transfers, debts, household size, cost of living, etcetera.

So, is there an income threshold at $100,000 (or some higher level) “beyond which the miseries that remain are not alleviated by high income,” where money can no longer “buy happiness”? For now, let’s keep shopping for answers.

## Data Availability

Materials and code at https://osf.io/txcrv/ (7).
